# Evaluation of the frequency of ABO and Rh-Hr blood-group systems in different acquired cataracts type

**DOI:** 10.1186/s13104-023-06524-7

**Published:** 2023-09-30

**Authors:** Reza Jafari, Hanieh Ahmadi, Samira Chaibakhsh, Fatemeh Rostamian Motlagh, Samira Heydarian, Ahmad Ahmadzadeh Amiri, Asadollah Farrokhfar, Ghasem Rostami, Mahdi Abounoori

**Affiliations:** 1 Department of Ophthalmology, Bu-Ali Sina Hospital, Mazandaran University of Medical Sciences, Sari, Iran; 2 Department of Ophthalmology, Eye Research Center, Eye Department, The Five Senses Health Institute, School of Medicine, Rassoul Akram Hospital, Iran University of Medical Sciences, Tehran, Iran; 3 Faculty of Medicine, Azad University of Mazandaran, Sari, Iran; 4 Department of Rehabilitation Sciences, School of Allied Medical Sciences, Mazandaran University of Medical Sciences, Sari, Iran; 5 Student Research Committee, School of Medicine, Mazandaran University of Medical Sciences, Sari, Iran; 6 Isfahan Eye Research Center, Isfahan University of Medical Sciences, Isfahan, Iran

**Keywords:** Cataract, ABO blood-group system, Rh-Hr blood-group system

## Abstract

**Objectives:**

This study evaluated the relationship between acquired cataract’s different types and the ABO and Rh blood classes.

**Methods:**

Overall, 520 patients, by randomized sampling method, participated in this retrospective cross-sectional study. After reviewing the patient’s medical records and laboratory results, the patient’s demographics, ABO group, Rh, and cataract type were documented.

**Results:**

A total of 520 patients were included in the research, with a mean age of 67.57 ± 11.85. Most of them were female (n = 286, 55%). Mix (n = 230, 44%) and nuclear sclerotic (NS) (n = 167, 32%) cataracts were the most common types. The posterior subcapsular cataract (PSC) prevalence in females was significantly higher than in males (16.1% vs.7.3% p = 0.002). Also, men had more NS cataracts than females (89, 38% vs. 78, 27.3%) (p = 0.009). Patients with PSC were significantly younger than others (all p-values < 0.001). Our results showed that cataract types are independent of blood group types and Rh (P > 0.05).

**Conclusion:**

Although our findings showed that cataract types are independent of blood group types and Rh, they can be compared with future studies on the association of other Blood-Group Systems in developing acquired cataracts.

## Introduction

A cataract is a lens abnormality characterized by increasing cloudiness and reduced clarity, and globally, cataract is the most common factor in reversible vision impairment and blindness [1]. The primary proteins comprising the lens and the lens surfaces, crystallins, give lenses their refractive properties. The primary processes behind the formation of cataracts include crystallin modification, aggregation, and precipitation. There is currently no known way to stop this procedure. Most cataracts are brought on by age-related degeneration, but they may also appear due to trauma or underlying disease [2].

The ABO Blood-Group System, which is thought to be the most significant blood group system, has a wide distribution of antigens. Red blood cells, platelets, and organs like endothelial cells contain these antigens. Consequently, they are known as histo-blood group antigens. The Rh-Hr Blood-Group System’s D antigen is the second most significant after ABO antigens. Rh + people have D antigen, while Rh^−^ lack it [3].

Several blood group systems have long been recognized to be connected to life-threatening illnesses [4–6]. Relevant instances include the I (ISBT 027) blood group system, depending on the underlying mutation, may be accompanied by congenital cataracts [7].

It is generally known that environmental conditions and genetic predisposition are linked to cataract development [2]. However, no research has examined the potential connection between acquired cataracts’ different types and the ABO and Rh blood classes.

## Materials and methods

### Study design and population

Between April 2010 and December 2015, 520 patients, by randomized sampling method, participated in this retrospective cross-sectional study at the Department of Ophthalmology, Mzandaran University of Medical Sciences. The research was conducted following the guidelines of the Helsinki Declaration after receiving approval from the institutional ethics committee (Mazandaran University of Medical Sciences) (IR.MAZUMS.REC.1394.932). All patients involved in this report were given written informed consent before data collection.

Along with blood types and Rh factors, the patient’s demographics and the type of cataract were noted. After reviewing the patient’s medical records and laboratory results, blood groups for the patients were determined.

### Exclusion criteria

Those who had a history of using tobacco or alcohol, intraocular pressure (IOP) greater than 21 mmHg, any systemic disease (such as diabetes and hypertension), any glaucomatous findings (such as glaucomatous optic disk changes and visual field defects), a history of intraocular surgery or laser treatment, a history of contact lens wear, strabismus, ocular trauma, amblyopia, cataract, taking any medication within the previous three months and any ocular inflammatory disease were excluded.

### Ophthalmological examination

A slit-lamp microscope (Haag-Streit model BQ-900; Haag-Streit, Koeniz, Switzerland) was used to evaluate the lens opacities using the standardized Lens Opacities Classification System (LOCS) III photographic images. In comparison to photographic standards, lens characteristics were divided into four categories: nuclear sclerotic (NS, LOCS III score ≥ 4 for nuclear opalescence or ≥ 4 for nuclear color), cortical (LOCS III score ≥ 4 for cortical cataract), posterior subcapsular (PSC, LOCS III score ≥ 2 for PSC cataract), and mixed cataract (more than one type in an eye). One of the five forms of cataract in at least one eye was considered to be any cataract.

### Statical analysis

Data were described by mean (SD) or frequency (%). For Comparing the mean in two groups, an independent t-test was performed, and for comparing the mean of more than two groups, analysis of variance was used. Post-hoc comparisons were made using the Tukey method. The chi-square or Fisher exact test was used to find the relation between categorical variables. All the analyses were utilized by SPSS 25. p-value < 0.05 was considered as the significant level.

## Results

A total of 520 patients were included in the research, with a mean age of 67.57 ± 11.85 ranging from 27 to 94. Most of them were female (n = 286, 55%). Among different types of cataracts, Mix was the most frequent type (n = 230, 44%), followed by NS (n = 167, 32%).

The cortical (35, 12.2% vs. 25, 11%), mix (127, 44.4% vs.103, 44%), and PSC (46, 16.1% vs. 17, 7.3%) types were more prevalent in females than males. The female-to-male predominance of cortical, cix, and PSC cataracts was significant (P = 0.002) only in the PSC type. Conversely, males significantly (P = 0.009) had more NS cataract frequency than females (89, 38% vs. 78, 27.3%) (Table 1).

**Table 1:** Type of Cataract and gender

Cataract type	Female, n (%)	Male, n (%)	*P- value*
**Cortical** **Other**	35 (12.2) 251 (87.76)	25 (11) 209 (89.32)	0.581
**Mix** **Other**	127 (44.4) 159 (55.6)	103 (44) 131 (56)	0.929
**NS** **Other**	78 (27.3) 208 (72.7)	89 (38) 145 (62)	0.009
**PSC** **Other**	46 (16.1) 240 (83.9)	17 (7.3) 217 (92.7)	0.002

The mean age in patients with cortical, mix, NS, and PSC types were 66.65 ± 15.05, 68.89 ± 10.75, 69.97 ± 10.44, and 56.60 ± 10.03, receptively. The mean age was significantly lower in PSC compared to all other types (all Ps < 0.001). There was no significant difference between other comparisons regarding age (Table 2).

**Table 2:** Type of Cataract and age

Cataract type	Age, mean ± SD	*P- value*
**Cortical**	66.65 ± 15.05	Vs. Mix: >0.999 Vs. NS: 0.384 Vs. PSC: <0.001
**Mix**	68.89 ± 10.75	Vs. Cortical: >0.999 Vs. NS: >0.999 Vs. PSC: <0.001
**NS**	69.97 ± 10.44	Vs. Cortical: 0.384 Vs. Mix: >0.999 Vs. PSC: <0.001
**PSC**	56.60 ± 10.03	For all of the comparisons: <0.001

Among the included patients, 159 had A, 38 had AB, 116 had B, and 196 had O blood group. No significant differences were observed when each blood group in each cataract type was compared to other blood group types (P > 0.05) (Table 3). Also, there was no significant relation between Rh and the type of cataract (P > 0.05) (Table 4). So, the prevalences of cataract types were the same in the case of different blood types and Rh. For further evaluation, a power analysis was done. The powers of the tests changed from 71 to 85%.

**Table 3:** Type of Cataract and ABO blood group

Cataract type	A, n (%)	AB, n (%)	B, n (%)	O, n (%)	*P- value*
**Cortical** **Other**	15 (9.4) 144 (90.6)	6 (15.8) 32 (84.2)	14 (11.1) 112 (88.9)	25 (12.8) 171 (87.2)	0.644
**Mix** **Other**	78 (49.1) 81 (50.9)	16 (42.1) 22 (57.9)	47 (37.3) 79 (62.7)	88 (44.9) 108 (55.1)	0.255
**NS** **Other**	48 (30.2) 111 (69.8)	12 (31.6) 26 (68.4)	45 (35.7) 81 (64.3)	62 (31.6) 134 (68.4)	0.791
**PSC** **Other**	18 (11.3) 141 (88.7)	4 (10.5) 34 (89.5)	20 (15.9) 106 (84.1)	21 (10.7) 175 (89.3)	0.529
**Total**	159 (100)	38 (100)	116 (100)	196 (100)	

**Table 4:** Type of Cataract and Rh

Cataract type	Rh -, n (%)	Rh +, n (%)	*P- value*
**Cortical** **Other**	6 (20.7) 23 (79.3)	54 (11) 436 (89)	0.130
**Mix** **Other**	10 (34.5) 19 (65.5)	219 (44.7) 271 (55.3)	0.282
**NS** **Other**	8 (27.6) 21 (72.4)	159 (32.4) 331 (67.6)	0.586
**PSC** **Other**	5 (17.2) 24 (82.8)	58 (11.8) 432 (88.2)	0.379

## Discussion

This study’s main findings have revealed that the frequency of ABO and Rh blood group systems in each cataract type didn’t differ significantly from the others. The mix type was the most frequent. Of 520 patients, females had a higher prevalence of cortical, mix, and PSC cataracts than men. However, in terms of statistics, only the prevalence of PSC was statistically considerably greater in females, whereas differences in the prevalence of mix and cortical cataracts were not statistically significant. Also, NS cataract was more prevalent in men, which was statistically significant. Mean age significantly differed in NS, mix, and PSC compared to other groups. The mean age in PSC was significantly lower than others.

In the late 1800s, Mendelian inheritance of cataracts was reported. In the 1960s, an inherited form of cataract (CAE1) that was tightly connected with the Duffy blood-group locus (Fy) was discovered [8]. Also, the linkage of the relevant gene locus to the ABO, MNS, Rhesus, P, Kell, or Secretor loci is ruled out [9]. A later study found that depending on the underlying mutation, the congenital cataract may accompany the adult I phenotype in the I (ISBT 027) blood group system [10]. Still, in the setting of acquired cataracts, there is a gap in the literature about the linkage of blood groups and cataracts. This study demonstrated no linkage between the type of cataract with ABO blood groups and Rh factors in adults with acquired cataracts.

In a systematic review and meta-analysis study on the global prevalence of age-related cataracts, the estimated pooled prevalence of any cataract ranged from 3% in the 20- to 39-year age group to 54% in the over-60 age group. Other cataract forms, including nuclear, cortical, and PSC, have shown an increasing tendency of age-related cataracts (11). In this study, similar to our results, the mean age of the PSC type compared to all other types was significantly lower than 60 years old, but the other form’s mean age was over 60 years old. Other studies have found a rise in the occurrence of cataracts with age, commonly regarded as a natural component of the aging process [12–14].

We found that the cortical, mix, and PSC cataracts were more prevalent in females than males. Regardless of the criterion employed, most studies show that women have a higher prevalence of cataracts than men [15, 16]. It has been proposed that the drop in estrogen during menopause increases the risk of cataracts in women, i.e., the withdrawal impact rather than the concentration of estrogen [17]. Since oxidative stress is thought to play a significant role in cataractogenesis, researchers have looked into how estrogens affect lens epithelial cells in culture or animal models and discovered that physiological amounts of 17β-estradiol protect H2O2-induced oxidative stress in cultivated lens epithelial cells [17–19].

The 4-year incidence of lens opacities was assessed in the Barbados eye study. Age, female gender, poor socioeconomic level, and diabetes mellitus increased the risk of cortical opacity, posterior subcapsular opacity, and nuclear opacity, respectively [20].

Although there is a higher prevalence of nuclear sclerosing cataracts in female genders in different studies [20, 21], our study is similar to a systematic review and meta-analysis study on the global and regional prevalence of age-related cataracts [11], demonstrated this subtype is more prevalent in males. It could be due to different sample sizes, age groups, and global regions [11].

## Conclusion

This study’s main findings have revealed no relationship between ABO and Rh blood groups and different types of acquired cataracts in adults. The findings can be compared with future studies on the association of other Blood-Group Systems in developing acquired cataracts.

## Limitations

The lack of determining other blood group systems’ association with cataracts was one of this study’s limitations.

## Data Availability

The data sets generated for this study are available at reasonable request to the corresponding author.
